# Multisystem FDG-avid lesions mimicking malignancy in a teenager with hyper-IgE syndrome: A case study with literature review

**DOI:** 10.22038/aojnmb.2026.91014.1671

**Published:** 2026

**Authors:** Fatemeh Saboktakin, Saeed Farzanefar, Nasim Vahidfar, Shaghayegh Ranjbar, Niloofar Tabatabaeian

**Affiliations:** Department of Nuclear Medicine, Vali-Asr Hospital, Tehran University of Medical Sciences, Tehran, Iran

**Keywords:** Hyper-IgE syndrome, Job’s syndrome, FDG-PET

## Abstract

Hyper-IgE Syndrome (HIES) is a rare immunodeficiency predisposing patients to infections, inflammation, and occasional neoplasia. We report a 17-year-old boy with HIES who presented with severe right thigh pain. FDG-PET/CT demonstrated a right adrenal mass (SUV_max_=5.4), multiple pulmonary nodules, and a lytic-sclerotic femoral lesion (SUV_max_=4.6), initially suggesting disseminated malignancy. Biopsy confirmed acute osteomyelitis and a benign spindle cell tumor of the adrenal gland. Two months later, persistent fever and elevated ESR prompted re-evaluation, revealing intense FDG uptake in the thoracic aorta (SUV_max_=20.5) consistent with large vessel vasculitis, later confirmed by angiography. The combination of infection, benign tumor, and vasculitis illustrates the broad FDG uptake spectrum in HIES and the risk of misdiagnosis as malignancy. Integration of clinical, imaging, and histopathologic data was critical for accurate diagnosis. This case highlights FDG-PET/CT’s value in detecting inflammatory vascular disease and monitoring therapy, while reinforcing the importance of biopsy for definitive diagnosis. In immunodeficient patients, a multimodal diagnostic approach is essential to distinguish between malignant and benign FDG-avid lesions.

## Introduction

 Hyper-IgE Syndrome (HIES), or Job’s Syndrome, is a rare immunodeficiency characterized by high serum IgE, eosinophilia, eczema, and recurrent staphylococcal infections (1). It is commonly associated with STAT3 mutations and has systemic manifestations including pulmonary infections, skeletal abnormalities, and vasculopathies (2). Due to dysregulated immunity, HIES patients may develop atypical inflammatory lesions that mimic malignancy on functional imaging such as ^18^F-FDG PET/CT (3). This poses a diagnostic dilemma, as FDG uptake is not specific to neoplasia but may reflect infection, inflammation, or immune activation (4).

## Case Presentation

 A 17-year-old boy with known hyper-IgE syndrome (HIES) presented with right thigh pain, fever, and fatigue. His history included recurrent bacterial infections and atopic dermatitis.

**Figure 1:**
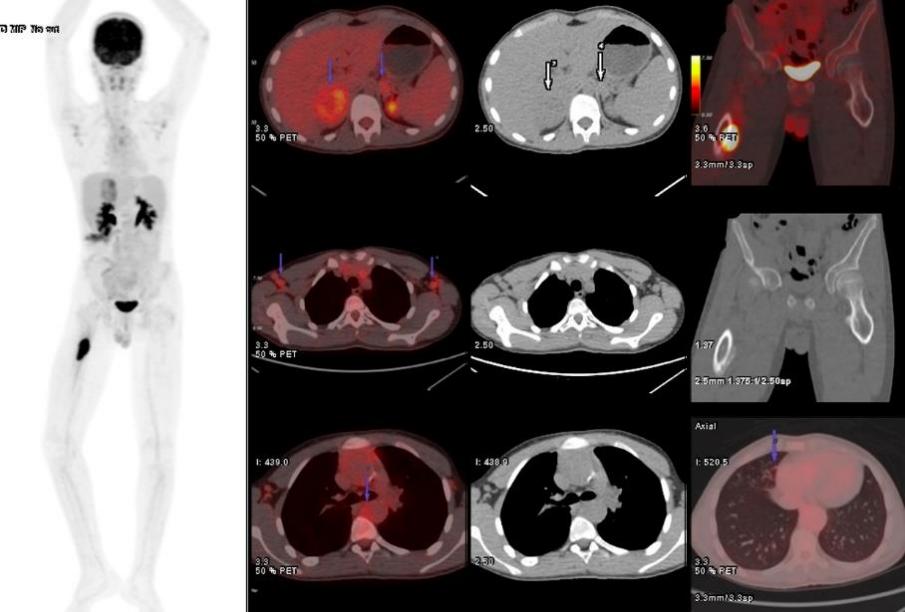
17 y/o patient with history of HIES underwent PET/CT scan due to right femoral pain. The study is suggestive of FDG avid right adrenal mass (measuring 51×55mm, SUV_max_= 5.4), mildly FDG avid left adrenal nodule (measuring 18×12mm, SUV_max_=2.7) and few pulmonary nodules with mild FDG uptake, largest one located in RML (measuring 6mm, SUV_max_=1.4), hypermetabolic lytic sclerotic right femoral osseous lesion along with periosteal reaction (SUV_max_=4.6) and multiple hypermetabolic bilateral axillary lymph nodes, some with thick cortex (measuring up to 12×12mm, SUV_max _up to 2.9)

Initial FDG-PET/CT (figure 1) findings included:

- FDG-avid right adrenal mass (51×55 mm, SUV_max_=5.4)
- Small mildly FDG-avid left adrenal nodule
- Multiple FDG-avid pulmonary nodules
- Lytic-sclerotic lesion in the right femur with periosteal reaction (SUV_max_=4.6), suspicious for malignant bone lesion (more in favor of Ewing sarcoma).

 These findings suggested a malignant bone tumor with possible adrenal and pulmonary metastases. Biopsies were performed on the right femur and adrenal mass.

Histopathology results:

- Right femur: Acute osteomyelitis with dense neutrophilic infiltration, edema, vascular congestion, and early osteocyte necrosis.
- Right adrenal mass: Leiomyoma composed of spindle cells with uniform nuclei, eosinophilic cytoplasm, no mitotic activity, and chronic inflammatory infiltrates in hyalinized and edematous stroma. IHC showed h-caldesmon and desmin positivity (smooth muscle origin), with negative ALK, SOX10, Myogenin, and MyoD1. Ki-67 index was ~3-4%.

The patient improved with antibiotic therapy for osteomyelitis. However, two months later, persistent fever, elevated ESR, and echocardio-graphic findings (LVEF=50-55%, turbulent flow) raised suspicion for vasculitis.

**Figure 2:**
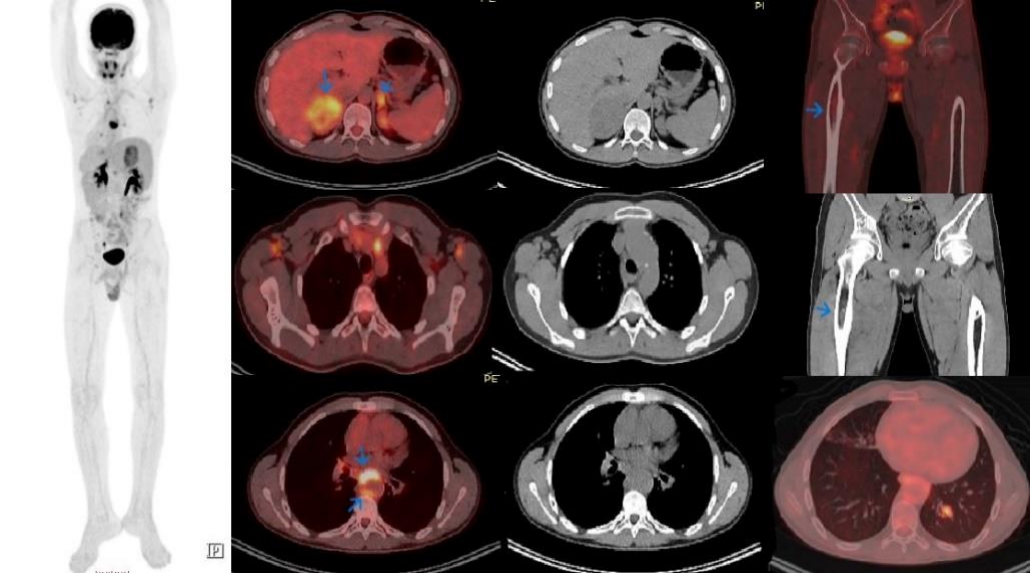
PET/CT Follow-up scan reveals FDG avid right adrenal mass (measuring 40×42mm, SUV_max_=5.2) and mildly FDG avid left adrenal nodule which are stable since previous PET study. Previously noted pulmonary nodule in RML resolved in current study and a new pulmonary nodule appears in the LLL (measuring 13mm, SUV_max_=4). Interval developed semi-circumferential intense FDG uptake (SUV_max_=20.5) is seen in the anterior descending thoracic aorta (10 to 1 o’clock below the carina), circumferential intermediate-grade uptake (SUV_max_=2.3) along the remaining thoracic aorta is also noted. Previously noted hypermetabolic axilla lymph nodes are again noted which are stable since previous study. Also, previously noted FDG-avid right femoral lesion now appeared as non-FDG-avid cortical thickening in the midshaft of femoral diaphysis, consistent with chronic or treated osteomyelitis

 Follow-up PET/CT (Figure 2) findings:

- Semi-circumferential intense FDG uptake (SUV_max_=20.5) in the anterior descending thoracic aorta (10 to 1 o’clock below the carina)
- Circumferential intermediate-grade uptake (SUV_max_=2.3) along the remaining thoracic aorta-grade 2 compared to liver-suggestive of large vessel vasculitis
- Previously FDG-avid right femoral lesion now appeared as non-avid cortical thickening of the midshaft femoral diaphysis, consistent with chronic or treated osteomyelitis

Angiography confirmed post-subclavian coarctation and diffuse aortic wall thickening, consistent with large vessel vasculitis.

## Discussion

 This case illustrates the diagnostic challenges of interpreting FDG-PET/CT in primary immunodeficiency syndromes such as hyper-IgE syndrome (HIES), where increased FDG uptake may reflect malignancy, infection, inflammation, or benign tumors (5,6). In this patient, the initial FDG-PET/CT revealed a suspicious bone lesion and adrenal mass. Due to persistent fever and elevated ESR, a follow-up scan identified large vessel vasculitis. Final diagnoses included acute osteomyelitis, adrenal leiomyoma, and Takayasu-like vasculitis-confirmed via echocardiography, angiography, and histopathology.

 Histology of the adrenal mass showed a benign spindle cell tumor with no mitotic activity, embedded in hyalinized and edematous stroma with chronic inflammation. IHC confirmed smooth muscle origin (positive for h-caldesmon, desmin) and excluded IMT (ALK-), schwannoma (SOX10-), and rhabdo-myosarcoma (Myogenin-, MyoD1-). A low Ki-67 index (3–4%) supported the diagnosis of leiomyoma, a rare adrenal tumor likely from vascular smooth muscle (7–9). The bone lesion, initially suggestive of malignancy (e.g., Ewing sarcoma), was confirmed as acute osteomyelitis, consistent with HIES-related infection susceptibility (10).

 Vasculitis was suspected due to persistent fever, elevated ESR, and echocardiographic signs of turbulent flow (LVEF=50-55%). 

 PET/CT showed focal increased FDG uptake in the thoracic aorta (SUV_max_=20.5 anteriorly, 2.3 elsewhere), suggestive of vasculitis (11). Angiography revealed post-subclavian coarctation and diffuse aortic thickening, confirming the diagnosis (12).

 HIES, often caused by STAT3 mutations, and predisposes to infections, connective tissue anomalies, and rare vasculitis (13). Freeman et al. emphasized the role of dysregulated cytokines in osteomyelitis, vasculitis, and chronic inflammation in benign tumors (3). This triad of lesions reflects immune dysregulation and complicates FDG interpretation.

 Although highly sensitive, FDG-PET/CT lacks specificity in immunocompromised hosts, where infection, inflammation, and benign lesions may mimic cancer (4). Sathekge et al. reported 85–90% sensitivity but only 50% specificity in such settings (14). The adrenal leiomyoma’s diagnosis was supported by IHC markers distinguishing it from sarcomas (15, 16). Similarly, Ewing sarcoma and osteomyelitis, with overlapping imaging features, require biopsy for differentiation (17). 

 PET/CT also proved valuable in assessing vasculitis, with Sağer et al. documenting high FDG uptake in Takayasu arteritis, resolving after treatment (18). Marco et al. advocated combining PET/CT and CT angiography to assess inflammation and vascular morphology simultaneously (19). Thus, integrating imaging, clinical, and pathological data was essential for accurate diagnosis and management in this complex HIES case.

## Conclusion

 In conclusion, this case highlights the complexity of interpreting FDG-PET/CT in HIES and similar conditions. The literature underscores the importance of recognizing benign mimics of malignancy, such as spindle cell tumors, and rare complications like vasculitis in HIES. Histological confirmation is critical, as metabolically active lesions in HIES may represent benign or inflammatory processes rather than malignancy. Also, clinicians must employ a multimodal diagnostic strategy to avoid misdiagnosis and guide appropriate management.
